# Binaural Heterophasic Superdirective Beamforming

**DOI:** 10.3390/s21010074

**Published:** 2020-12-25

**Authors:** Yuzhu Wang, Jingdong Chen, Jacob Benesty, Jilu Jin, Gongping Huang

**Affiliations:** 1 Center of Intelligent Acoustics and Immersive Communications, Northwestern Polytechnical University, 127 Youyi West Road, Xi’an 710072, China; im.yuzhu.wang@gmail.com (Y.W.); charles.jilu.jin@gmail.com (J.J.); 2 INRS-EMT, University of Quebec, 800 de la Gauchetiere Ouest, Montreal, QC H5A 1K6, Canada; benesty@emt.inrs.ca; 3 Andrew and Erna Viterby Faculty of Electrical Engineering, Technion-Israel Institute of Technology, Technion City, Haifa 32000, Israel; gongping@campus.technion.ac.il

**Keywords:** microphone arrays, binaural beamforming, heterophasic, superdirective beamformer, white noise gain, directivity factor, beampattern, interaural coherence

## Abstract

The superdirective beamformer, while attractive for processing broadband acoustic signals, often suffers from the problem of white noise amplification. So, its application requires well-designed acoustic arrays with sensors of extremely low self-noise level, which is difficult if not impossible to attain. In this paper, a new binaural superdirective beamformer is proposed, which is divided into two sub-beamformers. Based on studies and facts in psychoacoustics, these two filters are designed in such a way that they are orthogonal to each other to make the white noise components in the binaural beamforming outputs incoherent while maximizing the output interaural coherence of the diffuse noise, which is important for the brain to localize the sound source of interest. As a result, the signal of interest in the binaural superdirective beamformer’s outputs is in phase but the white noise components in the outputs are random phase, so the human auditory system can better separate the acoustic signal of interest from white noise by listening to the outputs of the proposed approach. Experimental results show that the derived binaural superdirective beamformer is superior to its conventional monaural counterpart.

## 1. Introduction

Microphone arrays combined with proper beamforming methods have been used in a wide range of applications, such as hearing aids, smart headphones, smart speakers, voice communication, automatic speech recognition (ASR), human–machine interfaces, etc., to extract signals of interest from noisy observations. Many beamformers have been developed over the last few decades [1,2,3,4,5,6,7,8,9,10,11,12,13,14,15,16,17], among which the so-called superdirective beamformer [9] is particularly attractive. It is derived by maximizing the directivity factor (DF), which is equivalent to maximizing the gain in signal-to-noise ratio (SNR) in diffuse noise, subject to the distortionless constraint at the endfire direction. So, this beamformer is more efficient than other fixed beamformers for suppressing noise, interference, and reflections incident from different directions in noisy and reverberant environments [14,18]. It also has a frequency-invariant beampattern if the sensor spacing is small, which is essential for acquiring high-fidelity broadband acoustic and speech signals.

However, there is one major drawback with the existing superdirective beamforming approach: the white noise amplification is very serious at low frequencies. As a consequence, the application of this beamformer requires well-designed arrays with high-quality microphones of extremely low self-noise level, e.g., at least below 0 dB(A) for second- and higher-order superdirective beamformers, which is difficult if not impossible to attain. This white noise amplification problem considerably limits the use of the superdirective beamformer in practical applications [18,19] and how to deal with this problem has become an important issue that has attracted a significant amount of research attention. A number of methods have been developed subsequently in the literature, including the so-called robust superdirective beamformer [9,20], the combined superdirective beamformer [21], the optimized superdirective beamformer [22], the subspace superdirective beamformer [11,23], and the reduced-rank superdirective beamformer [24]. While the problem is approached from different perspectives, the fundamental principle underlying those methods stays the same, i.e., making a compromise between the DF and the level of white noise gain (WNG) [10,20,22,25,26]. In other words, all those methods attempt to circumvent the white noise amplification problem by sacrificing the DF and, as a consequence, the resulting beamformer may no longer be superdirective.

In this work, we take a different avenue. Instead of sacrificing the DF to improve the WNG, we design the superdirective beamformer, which consists of two sub-beamformers, each generates an output. The two sub-beamformers are designed to be orthogonal to each other so that the acoustic signal of interest in the binaural outputs is in phase while the (amplified) white noise is random phase. This design is strongly motivated by studies and facts in psychoacoustics, which showed that the location (or direction) information of signals has a significant impact on speech intelligibility in the human auditory system.

**Figure 1:**
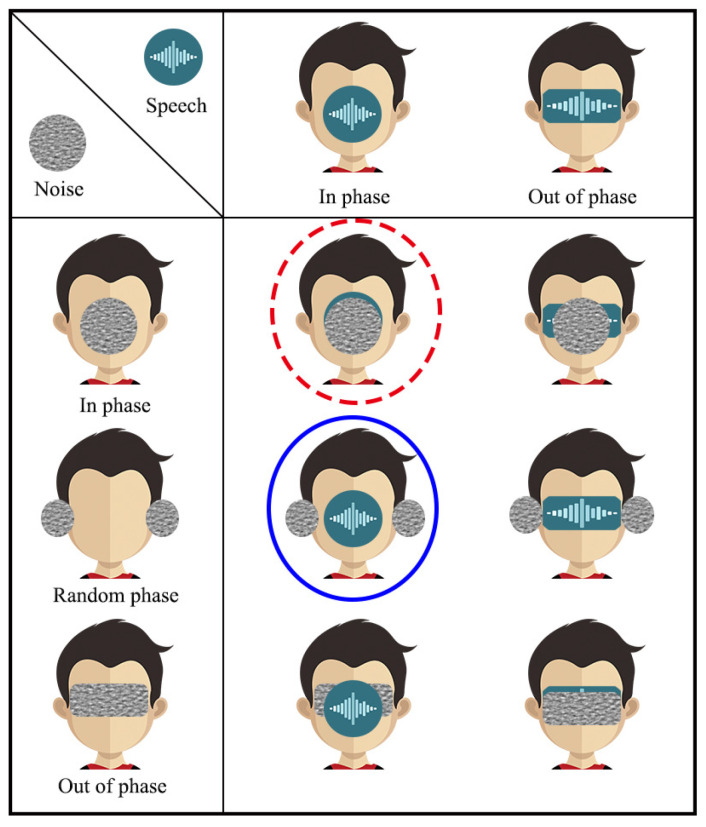
Illustration of different phase scenarios and the influence of the interaural phase relations on the localization of speech and white noise in space. The circle shape means that the signal is concentrated in a limited area, in front of the head for the case of in phase or on either side of the ears for the case of random phase. The rectangle shape indicates that the signal spreads in the area behind the ears, which is related to the case of out of phase. The red dotted circle indicates that the speech signal and the white noise are both in phase, which is related to the monaural superdirective beamformer. The blue circle indicates that the speech signal is in phase while the white noise is in random phase, which is related to the binaural superdirective beamformer developed in this work. This figure is a modified version of the results in [49].

Many experiments have been conducted to study the influence of the direction information of speech and white noise (especially at frequencies below 1 kHz) on speech intelligibility [27,28,29,30,31,32,33,34,35,36,37,38,39,40,41,42,43,44,45,46,47,48]. Briefly, the impact of the source direction on the perception of speech in the human binaural auditory system can be classified into two scenarios: in phase and out of phase; while the perception of noise can be divided into three scenarios: in phase, random phase, and out of phase, where in phase means that in every frequency the binaural (two-channel) signals have the same phase and out of phase means that in every frequency the phase difference between the binaural signals is exactly $180^{\circ }$. An illustration of the impact of binaural signals and noise phase on speech intelligibility, inspired from [49], is shown in Figure 1, where the leftmost column indicates the phase relationship of the white noise at the left and right channels, and the first row represents the phase relationship of binaural speech signals. The combination indicates the influence of interaural direction relations on the localization of speech and white noise in space. The circle shape indicates that the signal is concentrated in a limited area, in front of the head for the case of in phase or on either side of the ears for the case of random phase. The rectangle shape indicates that the signal spreads in the area behind both ears in the case of out of phase.

The blue circle indicates that the speech signals at the left and right channels are in phase but the white noise components at the two channels are in random phase, which is related to the proposed binaural superdirective beamformer.

Listening tests have been conducted to study the intelligibility of different phase combinations. It is confirmed that the phase combination of the desired speech signal and white noise has a significant influence on intelligibility. A list of the most common six scenarios, summarized in [49] and represented in Table 1, can be divided into three categories: antiphasic, heterophasic, and homophasic. Listening tests showed that the antiphasic category corresponds to the highest intelligibility, which can be 25% higher than that of the homophasic category in low SNR scenarios. The intelligibility of the heterophasic category is also higher than that of the homophasic case though lower than that of the antiphasic scenario. Inspired by this, it is desirable to design beamformers that make the binaural outputs corresponding to the antiphasic or heterophasic cases.

**Figure 2:**
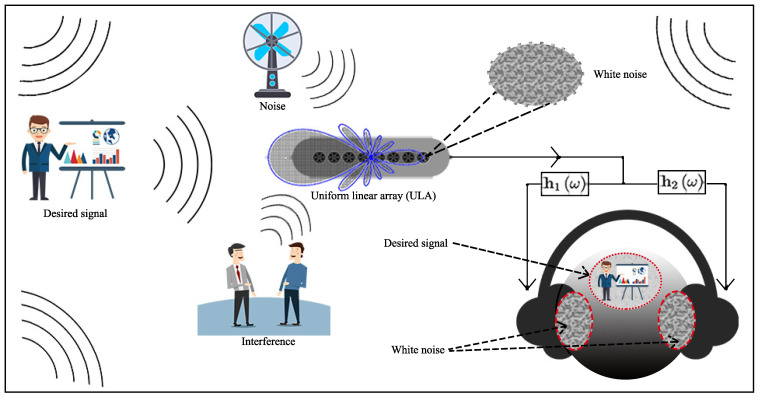
Illustration of the proposed binaural superdirective beamformer, which suppresses directional acoustic interference and noise and meanwhile separates the desired signal and the white noise into different zones.

In this paper, we use the interaural coherence (IC) to describe the auditory localization information. Consider a diffuse noise field; when the IC of the binaural signals reaches its maximum, i.e., 1, there is a precise region of the sound source, which is located in the middle of the head, i.e., the in-phase case; however, when the binaural signals are completely incoherent, i.e., the IC equals to 0, there are two independent sources at the two ears. This corresponds to the random phase case. In many approaches in the literature, binaural processing generates two collinear filters. The resulting output ICs for white (due to noise amplification) and diffuse noises are 1. So, both the signal of interest and the noise in the human auditory system are perceived to be in the same region. Consequently, our brain will have difficulties to separate them with a binaural presentation and intelligibility will certainly be affected. Apparently, the conventional monaural superdirective beamformer and the conventional collinear binaural processing belong to the homophasic case, which has the lowest intelligibility. To improve intelligibility, we propose a binaural superdirective beamformer by constructing two orthogonal filters, with which the IC for the white noise components is equal to zero while the IC for the desired signal components is equal to one in the binaural outputs. Consequently, with the proposed method, our auditory system can more easily distinguish between the signal of interest and the (amplified) white noise by listening to the binaural outputs, leading to improved intelligibility [50]. An illustration of the proposed binaural superdirective beamformer with a uniform linear array is shown in Figure 2, which can suppress spatial noise while seperating the desired signal and the white noise into different perception zone. Since the binaural superdirective beamformer developed in this paper corresponds to the heterophasic case [49] (see also Table 1), we name it binaural heterophasic superdirective beamformer. Note that the IC information has been used in the traditional binaural speech enhancement methods for binaural cues preservation [51,52]. However, the proposed binaural heterophasic superdirective beamformer uses the IC magnitude information in a very different way where the two orthogonal sub-beamformers are designed to minimize the IC magnitude of the white noise components while maximizing the IC magnitude of the diffuse noise components in the binaural outputs to achieve better perceptual separation of the signal of interest and white noise.

The rest of this paper is organized as follows. In Section 2, we present the signal model and formulate the problem. In Section 3, we briefly review the derivation of the conventional superdirective beamformer. In Section 4, we discuss binaural linear filtering and the associated performance measures. In Section 5, we derive the binaural heterophasic superdirective beamformer. Then, in Section 6, we present some experiments to validate the theoretical study. Finally, conclusions are presented in Section 7.

## 2. Signal Model and Problem Formulation

We consider a source signal of interest (plane wave), in the farfield, that propagates from the azimuth angle, θ, in an anechoic acoustic environment at the speed of sound, i.e., c=340m/s, and impinges on a uniform linear array (ULA) consisting of 2M omnidirectional microphones. In this scenario, the corresponding steering vector (of length 2M) is [5]

(1) $$\begin{array}{c} d\left(\omega ,\theta \right)={\left[\begin{array}{cccc} 1 & e^{-j\omega \tau_{0}\cos\theta } & \cdots & e^{-j\left(2M-1\right)\omega \tau_{0}\cos\theta } \end{array}\right]}^{T}, \end{array}$$

where *j* is the imaginary unit with $j^{2}=-1$, ω=2πf is the angular frequency, with f>0 being the temporal frequency, $\tau_{0}=\delta /c$ is the delay between two successive sensors at the angle θ=0, with δ being the interelement spacing, and the superscript ${}^{T}$ is the transpose operator.

Assume that the desired signal comes from a specific direction $\theta =\theta_{s}$. From the steering vector defined in (1), we can express the frequency-domain observation signal vector of length 2M as [2]

(2) $$\begin{array}{ccc} y\left(\omega \right) & = & {\left[\begin{array}{cccc} Y_{1}\left(\omega \right) & Y_{2}\left(\omega \right) & \cdots & Y_{2M}\left(\omega \right) \end{array}\right]}^{T}\\ & = & x\left(\omega \right)+v\left(\omega \right)\\ & = & d\left(\omega ,\theta_{s}\right)X\left(\omega \right)+v\left(\omega \right), \end{array}$$

where $Y_{m}\left(\omega \right)$ is the *m*th microphone signal, $x\left(\omega \right)=d\left(\omega ,\theta_{s}\right)X\left(\omega \right)$, $X\left(\omega \right)$ is the zero-mean source signal of interest, which is also called the desired signal, $d\left(\omega ,\theta_{s}\right)$ is the signal propagation vector, which is same as the steering vector at $\theta =\theta_{s}$, and $v\left(\omega \right)$ is the zero-mean additive noise signal vector defined similarly to $y\left(\omega \right)$. We deduce that the 2M×2M covariance matrix of $y\left(\omega \right)$ is

(3) $$\begin{array}{ccc} \Phi_{y}\left(\omega \right) & \overset{▵}{=} & E\left[y\left(\omega \right)y^{H}\left(\omega \right)\right]\\ & = & \phi_{X}\left(\omega \right)d\left(\omega ,\theta_{s}\right)d^{H}\left(\omega ,\theta_{s}\right)+\Phi_{v}\left(\omega \right)\\ & = & \phi_{X}\left(\omega \right)d\left(\omega ,\theta_{s}\right)d^{H}\left(\omega ,\theta_{s}\right)+\phi_{V_{1}}\left(\omega \right)\Gamma_{v}\left(\omega \right), \end{array}$$

where the superscript ${}^{H}$ is the conjugate-transpose operator, E[·] denotes mathematical expectation, $\phi_{X}\left(\omega \right)\overset{▵}{=}E\left[{\left|X\left(\omega \right)\right|}^{2}\right]$ is the variance of $X\left(\omega \right)$, $\Phi_{v}\left(\omega \right)\overset{▵}{=}E\left[v\left(\omega \right)v^{H}\left(\omega \right)\right]$ is the covariance matrix of $v\left(\omega \right)$, $\phi_{V_{1}}\left(\omega \right)\overset{▵}{=}E\left[{\left|V_{1}\left(\omega \right)\right|}^{2}\right]$ is the variance of the noise, $V_{1}\left(\omega \right)$, at the first sensor, and $\Gamma_{v}\left(\omega \right)\overset{▵}{=}\Phi_{v}\left(\omega \right)/\phi_{V_{1}}\left(\omega \right)$ is the pseudo-coherence matrix of the noise. We assume that noises at different sensors have the same variance.

In order to design the superdirective beamformer, we make two basic assumptions [9,19].

(i) The sensor spacing, δ, is much smaller than the acoustic wavelength, λ=c/f, i.e., δ≪λ (this implies that $\omega \tau_{0}\ll 2\pi$). This assumption is required so that the true acoustic pressure differentials can be approximated by finite differences of the microphones’ outputs.
(ii) The desired source signal propagates from the angle $\theta_{s}=0$ (endfire direction). Therefore, (2) becomes
  (4) $$\begin{array}{c} y\left(\omega \right)=d\left(\omega ,0\right)X\left(\omega \right)+v\left(\omega \right), \end{array}$$
  and, at the endfire, the value of the beamformer beampattern should always be equal to 1 (or maximal).

Our objective in this paper is to derive a binaural superdirective beamformer, which can take advantage of the human binaural auditory system to separate the desired speech signal from white noise so that the intelligibility of the beamformer’s output signals will be higher than that of the output of the conventional (monaural) superdirective beamformer. To that end, we will find two various and useful estimates of $X\left(\omega \right)$, each for one of the binaural channels, so that along with our binaural hearing system, white noise amplification will be perceptually attenuated thanks to this binaural presentation.

## 3. Conventional Superdirective Beamformer

The conventional linear fixed beamforming technique is performed by applying a complex weight at the output of each microphone and then sum all the weighted outputs together to get an estimate of the source signal [2,19], i.e.,

(5) $$\begin{array}{ccc} Z\left(\omega \right) & = & h^{H}\left(\omega \right)y\left(\omega \right)\\ & = & X\left(\omega \right)h^{H}\left(\omega \right)d\left(\omega ,0\right)+h^{H}\left(\omega \right)v\left(\omega \right), \end{array}$$

where $Z\left(\omega \right)$ is the estimate of the desired source signal, $X\left(\omega \right)$, and $h\left(\omega \right)$ is a spatial linear filter of length 2M containing all the complex weights. We see from (5) that the distortionless constraint should be

(6) $$\begin{array}{c} h^{H}\left(\omega \right)d\left(\omega ,0\right)=1. \end{array}$$

Now, we can define the directivity factor (DF) of the beamformer as [3,19,43]

(7) $$\begin{array}{ccc} D\left[h\left(\omega \right)\right] & \overset{▵}{=} & \frac{{\left|h^{H}\left(\omega \right)d\left(\omega ,0\right)\right|}^{2}}{\frac{1}{2}\int_{0}^{\pi }{\left|h^{H}\left(\omega \right)d\left(\omega ,\theta \right)\right|}^{2}\sin\theta d\theta }\\ & = & \frac{{\left|h^{H}\left(\omega \right)d\left(\omega ,0\right)\right|}^{2}}{h^{H}\left(\omega \right)\Gamma_{d}\left(\omega \right)h\left(\omega \right)}, \end{array}$$

where $\Gamma_{d}\left(\omega \right)=\frac{1}{2}\int_{0}^{\pi }d\left(\omega ,\theta \right)d^{H}\left(\omega ,\theta \right)\sin\theta d\theta$ whose ijth (i,j=1,2,…,2M) element is

(8) $$\begin{array}{ccc} {\left[\Gamma_{d}\left(\omega \right)\right]}_{ij} & = & \frac{\sin\left[\omega \left(j-i\right)\tau_{0}\right]}{\omega \left(j-i\right)\tau_{0}}\\ & = & \mathrm{sinc}\left[\omega \left(j-i\right)\tau_{0}\right]. \end{array}$$

The matrix $\Gamma_{d}\left(\omega \right)$ can be viewed as the pseudo-coherence matrix of the spherically isotropic (diffuse) noise.

By taking into account the distortionless constraint in (6), the maximization of the DF in (7) leads to the well-known superdirective beamformer [9,53]:

(9) $$\begin{array}{c} h_{\mathrm{SD}}\left(\omega \right)=\frac{\Gamma_{d}^{-1}\left(\omega \right)d\left(\omega ,0\right)}{d^{H}\left(\omega ,0\right)\Gamma_{d}^{-1}\left(\omega \right)d\left(\omega ,0\right)}, \end{array}$$

whose DF is

(10) $$\begin{array}{c} D\left[h_{\mathrm{SD}}\left(\omega \right)\right]=d^{H}\left(\omega ,0\right)\Gamma_{d}^{-1}\left(\omega \right)d\left(\omega ,0\right), \end{array}$$

which, obviously, is maximal. Besides maximizing the DF, the other great advantage of $h_{\mathrm{SD}}\left(\omega \right)$ is that the corresponding beampattern is almost frequency invariant. However, white noise amplification is a tremendous problem. Consequently, the superdirective beamformer can only be used with a very small number of microphones and/or with regularization of the matrix $\Gamma_{d}\left(\omega \right)$, but this regularization affects the DF as well as the shape of the beampattern, which makes the beamformer more frequency dependent. Therefore, there is still a great interest to find new ideas to improve this superdirective beamformer.

## 4. Binaural Linear Filtering and Performance Measures

In this section, we explain binaural linear filtering in connection with fixed beamforming and propose some important performance measures in this context.

The extension of the conventional (monaural) fixed linear beamforming to the binaural case can be done by applying two complex-valued linear filters, $h_{1}\left(\omega \right)$ and $h_{2}\left(\omega \right)$ of length 2M, to the observed signal vector, $y\left(\omega \right)$, i.e.,

(11) $$\begin{array}{ccc} Z_{i}\left(\omega \right) & = & h_{i}^{H}\left(\omega \right)y\left(\omega \right)\\ & = & X\left(\omega \right)h_{i}^{H}\left(\omega \right)d\left(\omega ,0\right)+h_{i}^{H}\left(\omega \right)v\left(\omega \right),\;i=1,2, \end{array}$$

where $Z_{1}\left(\omega \right)$ and $Z_{2}\left(\omega \right)$ are two different estimates of $X\left(\omega \right)$. The variance of $Z_{i}\left(\omega \right)$ is then

(12) $$\begin{array}{ccc} \phi_{Z_{i}}\left(\omega \right) & = & h_{i}^{H}\left(\omega \right)\Phi_{y}\left(\omega \right)h_{i}\left(\omega \right)\\ & = & \phi_{X}\left(\omega \right){\left|h_{i}^{H}\left(\omega \right)d\left(\omega ,0\right)\right|}^{2}+h_{i}^{H}\left(\omega \right)\Phi_{v}\left(\omega \right)h_{i}\left(\omega \right)\\ & = & \phi_{X}\left(\omega \right){\left|h_{i}^{H}\left(\omega \right)d\left(\omega ,0\right)\right|}^{2}+\phi_{V_{1}}\left(\omega \right)h_{i}^{H}\left(\omega \right)\Gamma_{v}\left(\omega \right)h_{i}\left(\omega \right). \end{array}$$

It is clear that the two distortionless constraints are

(13) $$\begin{array}{c} h_{i}^{H}\left(\omega \right)d\left(\omega ,0\right)=1,\;i=1,2. \end{array}$$

A very important performance measure is the input SNR, which can be obtained from (3), i.e.,

(14) $$\begin{array}{c} \mathrm{iSNR}\left(\omega \right)=\frac{\phi_{X}\left(\omega \right)}{\phi_{V_{1}}\left(\omega \right)}. \end{array}$$

According to (12), the binaural output SNR can be defined as

(15) $$\begin{array}{c} \mathrm{oSNR}\left[h_{1}\left(\omega \right),h_{2}\left(\omega \right)\right]=\frac{\phi_{X}\left(\omega \right)}{\phi_{V_{1}}\left(\omega \right)}\times \frac{\sum_{i=1}^{2}{\left|h_{i}^{H}\left(\omega \right)d\left(\omega ,0\right)\right|}^{2}}{\sum_{i=1}^{2}h_{i}^{H}\left(\omega \right)\Gamma_{v}\left(\omega \right)h_{i}\left(\omega \right)}. \end{array}$$

In the particular case of $h_{1}\left(\omega \right)=i_{i}$ and $h_{2}\left(\omega \right)=i_{j}$, the binaural output SNR is equal to the input SNR, in which $i_{i}$ and $i_{j}$ are, respectively, the *i*th and *j*th columns of $I_{2M}$ (i.e., the 2M×2M identity matrix). According to (14) and (15), the binaural SNR gain can be expressed as

(16) $$\begin{array}{ccc} G\left[h_{1}\left(\omega \right),h_{2}\left(\omega \right)\right] & = & \frac{\mathrm{oSNR}\left[h_{1}\left(\omega \right),h_{2}\left(\omega \right)\right]}{\mathrm{iSNR}\left(\omega \right)}\\ & = & \frac{\sum_{i=1}^{2}{\left|h_{i}^{H}\left(\omega \right)d\left(\omega ,0\right)\right|}^{2}}{\sum_{i=1}^{2}h_{i}^{H}\left(\omega \right)\Gamma_{v}\left(\omega \right)h_{i}\left(\omega \right)}. \end{array}$$

From the above definition, the following two measures that are very helpful for binaural fixed beamforming can be deduced:

- the binaural white noise gain (WNG):
  (17) $$\begin{array}{c} W\left[h_{1}\left(\omega \right),h_{2}\left(\omega \right)\right]=\frac{\sum_{i=1}^{2}{\left|h_{i}^{H}\left(\omega \right)d\left(\omega ,0\right)\right|}^{2}}{\sum_{i=1}^{2}h_{i}^{H}\left(\omega \right)h_{i}\left(\omega \right)} \end{array}$$
- and the binaural DF:
  (18) $$\begin{array}{c} D\left[h_{1}\left(\omega \right),h_{2}\left(\omega \right)\right]=\frac{\sum_{i=1}^{2}{\left|h_{i}^{H}\left(\omega \right)d\left(\omega ,0\right)\right|}^{2}}{\sum_{i=1}^{2}h_{i}^{H}\left(\omega \right)\Gamma_{d}\left(\omega \right)h_{i}\left(\omega \right)}, \end{array}$$
  where $\Gamma_{d}\left(\omega \right)$ is defined in the previous section.

The beampattern is another fundamental performance measure for fixed beamformers. The binaural beampattern can be defined as

(19) $$\begin{array}{c} {\left|B\left[h_{1}\left(\omega \right),h_{2}\left(\omega \right),\theta \right]\right|}^{2}=\frac{1}{2}\sum_{i=1}^{2}{\left|h_{i}^{H}\left(\omega \right)d\left(\omega ,\theta \right)\right|}^{2}. \end{array}$$

In order to have two meaningful estimates of the desired signal, we are going to extensively exploit the interaural coherence (IC) of the noise. It is well known that, in a multi-source environment, the IC (or its modulus) is important for source localization since it is very strongly related to the two principal binaural cues, i.e., the interaural time difference (ITD) and interaural level difference (ILD), that the brain uses to localize sounds. Psychoacoustically, the localization performance decreases when the IC decreases [33]. Furthermore, the IC affects significantly the perception for acoustic field width.

Let $A\left(\omega \right)$ and $B\left(\omega \right)$ be two zero-mean complex-valued random variables. The coherence function (CF) between $A\left(\omega \right)$ and $B\left(\omega \right)$ is defined as

(20) $$\begin{array}{c} \gamma_{AB}\left(\omega \right)=\frac{E\left[A\left(\omega \right)B^{*}\left(\omega \right)\right]}{\sqrt{E\left[{\left|A\left(\omega \right)\right|}^{2}\right]E\left[{\left|B\left(\omega \right)\right|}^{2}\right]}}, \end{array}$$

where the superscript ${}^{*}$ is the complex-conjugate operator. It is clear that $0\leq {\left|\gamma_{AB}\left(\omega \right)\right|}^{2}\leq 1$. For any pair of sensors (i,j), the input IC of the noise is simply the CF between $V_{i}\left(\omega \right)$ and $V_{j}\left(\omega \right)$, i.e.,

(21) $$\begin{array}{ccc} \gamma_{V_{i}V_{j}}\left(\omega \right) & = & \frac{E\left[V_{i}\left(\omega \right)V_{j}^{*}\left(\omega \right)\right]}{\sqrt{E\left[{\left|V_{i}\left(\omega \right)\right|}^{2}\right]E\left[{\left|V_{j}\left(\omega \right)\right|}^{2}\right]}}\\ & = & \frac{i_{i}^{T}\Phi_{v}\left(\omega \right)i_{j}}{\sqrt{i_{i}^{T}\Phi_{v}\left(\omega \right)i_{i}\times i_{j}^{T}\Phi_{v}\left(\omega \right)i_{j}}}\\ & = & \frac{i_{i}^{T}\Gamma_{v}\left(\omega \right)i_{j}}{\sqrt{i_{i}^{T}\Gamma_{v}\left(\omega \right)i_{i}\times i_{j}^{T}\Gamma_{v}\left(\omega \right)i_{j}}}\\ & = & \gamma \left[i_{i}\left(\omega \right),i_{j}\left(\omega \right)\right]. \end{array}$$

For white noise, the input IC is $\gamma_{w}\left(\omega \right)=0$, obviously. For diffuse noise, $\Gamma_{v}\left(\omega \right)=\Gamma_{d}\left(\omega \right)$, the input IC is ${\left[\Gamma_{d}\left(\omega \right)\right]}_{ij}$.

Similarly, we can define the output IC of the noise as the CF between the filtered noises in $Z_{1}\left(\omega \right)$ and $Z_{2}\left(\omega \right)$, i.e.,

(22) $$\begin{array}{ccc} \gamma \left[h_{1}\left(\omega \right),h_{2}\left(\omega \right)\right] & = & \frac{h_{1}^{H}\left(\omega \right)\Phi_{v}\left(\omega \right)h_{2}\left(\omega \right)}{\sqrt{h_{1}^{H}\left(\omega \right)\Phi_{v}\left(\omega \right)h_{1}\left(\omega \right)\times h_{2}^{H}\left(\omega \right)\Phi_{v}\left(\omega \right)h_{2}\left(\omega \right)}}\\ & = & \frac{h_{1}^{H}\left(\omega \right)\Gamma_{v}\left(\omega \right)h_{2}\left(\omega \right)}{\sqrt{h_{1}^{H}\left(\omega \right)\Gamma_{v}\left(\omega \right)h_{1}\left(\omega \right)\times h_{2}^{H}\left(\omega \right)\Gamma_{v}\left(\omega \right)h_{2}\left(\omega \right)}}. \end{array}$$

In the particular case of $h_{1}\left(\omega \right)=i_{i}$ and $h_{2}\left(\omega \right)=i_{j}$, the input and output ICs are equal, i.e., $\gamma \left[i_{i}\left(\omega \right),i_{j}\left(\omega \right)\right]=\gamma \left[h_{1}\left(\omega \right),h_{2}\left(\omega \right)\right]$. It can be checked that the output ICs of white (with the same power) and diffuse noises can be presented as, separately,

(23) $$\begin{array}{c} \gamma_{w}\left[h_{1}\left(\omega \right),h_{2}\left(\omega \right)\right]=\frac{h_{1}^{H}\left(\omega \right)h_{2}\left(\omega \right)}{\sqrt{h_{1}^{H}\left(\omega \right)h_{1}\left(\omega \right)\times h_{2}^{H}\left(\omega \right)h_{2}\left(\omega \right)}} \end{array}$$

and

(24) $$\begin{array}{c} \gamma_{d}\left[h_{1}\left(\omega \right),h_{2}\left(\omega \right)\right]=\frac{h_{1}^{H}\left(\omega \right)\Gamma_{d}\left(\omega \right)h_{2}\left(\omega \right)}{\sqrt{h_{1}^{H}\left(\omega \right)\Gamma_{d}\left(\omega \right)h_{1}\left(\omega \right)\times h_{2}^{H}\left(\omega \right)\Gamma_{d}\left(\omega \right)h_{2}\left(\omega \right)}}. \end{array}$$

In many approaches in the literature, the two derived filters $h_{1}\left(\omega \right)$ and $h_{2}\left(\omega \right)$ are collinear, i.e.,

(25) $$\begin{array}{cc} h_{1}\left(\omega \right) & =\varsigma \left(\omega \right)h_{2}\left(\omega \right), \end{array}$$

where $\varsigma \left(\omega \right)\neq 0$ is a complex-valued number. In this case, one can check that $\left|\gamma \left[h_{1}\left(\omega \right),h_{2}\left(\omega \right)\right]\right|=\left|\gamma_{w}\left[h_{1}\left(\omega \right),h_{2}\left(\omega \right)\right]\right|=\left|\gamma_{d}\left[h_{1}\left(\omega \right),h_{2}\left(\omega \right)\right]\right|=1$. Since the desired source signal is also fully coherent at any pair of sensors, both the desired signal and noise are perceived in the same region. As a result, our brain will have difficulties to separate them with binaural presentation and intelligibility will certainly be affected. For better separation between white noise and desired source, we should find orthogonal filters since, in this scenario, the output IC for white noise will be equal to 0, the same way is its corresponding input.

## 5. Binaural Heterophasic Superdirective Beamformer

In this section, we consider orthogonal binaural filters, i.e., $h_{1}^{H}\left(\omega \right)h_{2}\left(\omega \right)=0$, since we want the output IC for white noise to be zero. We also want to maximize $\gamma_{d}\left[h_{1}\left(\omega \right),h_{2}\left(\omega \right)\right]$ so that not only the signals of interest from a point source at the two binaural outputs are coherent, the diffuse (or any correlated) noise at the binaural outputs will be perceived as less diffuse as possible. For that, we will exploit the maximum modes of this CF.

The symmetric matrix $\Gamma_{d}\left(\omega \right)$ can be diagonalized as [54]

(26) $$\begin{array}{c} U^{T}\left(\omega \right)\Gamma_{d}\left(\omega \right)U\left(\omega \right)=\Lambda \left(\omega \right), \end{array}$$

where

(27) $$\begin{array}{c} U\left(\omega \right)=\left[\begin{array}{cccc} u_{1}\left(\omega \right) & u_{2}\left(\omega \right) & \cdots & u_{2M}\left(\omega \right) \end{array}\right] \end{array}$$

is an orthogonal matrix (note that each eigenvector is normalized according to the sign of its first value in our application because each eigenvector of $\Gamma_{d}\left(\omega \right)$ may have two opposite directions), i.e.,

(28) $$\begin{array}{c} U^{T}\left(\omega \right)U\left(\omega \right)=U\left(\omega \right)U^{T}\left(\omega \right)=I_{2M} \end{array}$$

and

(29) $$\begin{array}{c} \Lambda \left(\omega \right)=\mathrm{diag}\left[\lambda_{1}\left(\omega \right),\lambda_{2}\left(\omega \right),\ldots ,\lambda_{2M}\left(\omega \right)\right] \end{array}$$

is a diagonal matrix. The orthonormal vectors $u_{1}\left(\omega \right),u_{2}\left(\omega \right),\ldots ,u_{2M}\left(\omega \right)$ are the eigenvectors corresponding, respectively, to the eigenvalues $\lambda_{1}\left(\omega \right),\lambda_{2}\left(\omega \right),\ldots ,\lambda_{2M}\left(\omega \right)$ of the matrix $\Gamma_{d}\left(\omega \right)$, where $\lambda_{1}\left(\omega \right)\geq \lambda_{2}\left(\omega \right)\geq \cdots \geq \lambda_{2M}\left(\omega \right)>0$.

It can be shown that the two orthogonal filters that maximize (24) are [55]

(30) $$\left\{\begin{array}{c} h_{1}\left(\omega \right)=\frac{u_{1}\left(\omega \right)+u_{2M}\left(\omega \right)}{\sqrt{2}}=q_{+,1}\left(\omega \right)\\ h_{2}\left(\omega \right)=\frac{u_{1}\left(\omega \right)-u_{2M}\left(\omega \right)}{\sqrt{2}}=q_{-,1}\left(\omega \right) \end{array}\right..$$

In this case, we get the first maximum mode of the CF:

(31) $$\begin{array}{c} \gamma_{d}\left[q_{+,1}\left(\omega \right),q_{-,1}\left(\omega \right)\right]=\lambda_{\mp ,1}\left(\omega \right), \end{array}$$

with corresponding vectors $q_{+,1}\left(\omega \right)$ and $q_{-,1}\left(\omega \right)$, where

(32) $$\begin{array}{ccc} \lambda_{\mp ,1}\left(\omega \right) & = & \frac{\lambda_{1}\left(\omega \right)-\lambda_{2M}\left(\omega \right)}{\lambda_{1}\left(\omega \right)+\lambda_{2M}\left(\omega \right)}\\ & = & \frac{\lambda_{-,1}\left(\omega \right)}{\lambda_{+,1}\left(\omega \right)}. \end{array}$$

Similarly, we find that all the *M* maximum modes of the CF are

(33) $$\begin{array}{c} \gamma_{d}\left[q_{+,m}\left(\omega \right),q_{-,m}\left(\omega \right)\right]=\lambda_{\mp ,m}\left(\omega \right), \end{array}$$

for m=1,2,…,M, where

(34) $$\begin{array}{ccc} \lambda_{\mp ,m}\left(\omega \right) & = & \frac{\lambda_{m}\left(\omega \right)-\lambda_{2M-m+1}\left(\omega \right)}{\lambda_{m}\left(\omega \right)+\lambda_{2M-m+1}\left(\omega \right)}\\ & = & \frac{\lambda_{-,m}\left(\omega \right)}{\lambda_{+,m}\left(\omega \right)} \end{array}$$

and

(35) $$\begin{array}{c} \left\{\begin{array}{c} q_{+,m}\left(\omega \right)=\frac{u_{m}\left(\omega \right)+u_{2M-m+1}\left(\omega \right)}{\sqrt{2}}\\ q_{-,m}\left(\omega \right)=\frac{u_{m}\left(\omega \right)-u_{2M-m+1}\left(\omega \right)}{\sqrt{2}} \end{array}\right.. \end{array}$$

It can be verified that

(36) $$\begin{array}{c} \lambda_{\mp ,1}\left(\omega \right)\geq \lambda_{\mp ,2}\left(\omega \right)\geq \cdots \geq \lambda_{\mp ,M}\left(\omega \right). \end{array}$$

From (35), the two semi-orthogonal matrices (2M×M) can be written as

(37) $$\begin{array}{ccc} Q_{+}\left(\omega \right) & = & \left[\begin{array}{cccc} q_{+,1}\left(\omega \right) & q_{+,2}\left(\omega \right) & \cdots & q_{+,M}\left(\omega \right) \end{array}\right], \end{array}$$

(38) $$\begin{array}{ccc} Q_{-}\left(\omega \right) & = & \left[\begin{array}{cccc} q_{-,1}\left(\omega \right) & q_{-,2}\left(\omega \right) & \cdots & q_{-,M}\left(\omega \right) \end{array}\right], \end{array}$$

where

(39) $$\begin{array}{ccc} Q_{+}^{T}\left(\omega \right)Q_{+}\left(\omega \right) & = & Q_{-}^{T}\left(\omega \right)Q_{-}\left(\omega \right)=I_{M}, \end{array}$$

(40) $$\begin{array}{ccc} Q_{+}^{T}\left(\omega \right)Q_{-}\left(\omega \right) & = & Q_{-}^{T}\left(\omega \right)Q_{+}\left(\omega \right)=0, \end{array}$$

with $I_{M}$ being the M×M identity matrix. It can be shown that

(41) $$\begin{array}{ccc} Q_{+}^{T}\left(\omega \right)\Gamma_{d}\left(\omega \right)Q_{-}\left(\omega \right) & = & Q_{-}^{T}\left(\omega \right)\Gamma_{d}\left(\omega \right)Q_{+}\left(\omega \right)\\ & = & \Lambda_{-}\left(\omega \right), \end{array}$$

(42) $$\begin{array}{ccc} Q_{+}^{T}\left(\omega \right)\Gamma_{d}\left(\omega \right)Q_{+}\left(\omega \right) & = & Q_{-}^{T}\left(\omega \right)\Gamma_{d}\left(\omega \right)Q_{-}\left(\omega \right)\\ & = & \Lambda_{+}\left(\omega \right), \end{array}$$

where

(43) $$\begin{array}{ccc} \Lambda_{-}\left(\omega \right) & = & \frac{1}{2}\mathrm{diag}\left[\lambda_{-,1}\left(\omega \right),\lambda_{-,2}\left(\omega \right),\ldots ,\lambda_{-,M}\left(\omega \right)\right], \end{array}$$

(44) $$\begin{array}{ccc} \Lambda_{+}\left(\omega \right) & = & \frac{1}{2}\mathrm{diag}\left[\lambda_{+,1}\left(\omega \right),\lambda_{+,2}\left(\omega \right),\ldots ,\lambda_{+,M}\left(\omega \right)\right] \end{array}$$

are two diagonal matrices of size M×M.

Let *N* be a positive integer number with 2≤N≤M (a different value of *N* gives a different degree of tradeoff between WNG and DF). We define the two semi-orthogonal matrices (of size 2M×N):

(45) $$\begin{array}{ccc} Q_{+,:N}\left(\omega \right) & = & \left[\begin{array}{cccc} q_{+,1}\left(\omega \right) & q_{+,2}\left(\omega \right) & \cdots & q_{+,N}\left(\omega \right) \end{array}\right], \end{array}$$

(46) $$\begin{array}{ccc} Q_{-,:N}\left(\omega \right) & = & \left[\begin{array}{cccc} q_{-,1}\left(\omega \right) & q_{-,2}\left(\omega \right) & \cdots & q_{-,N}\left(\omega \right) \end{array}\right]. \end{array}$$

In the rest, we consider orthogonal filters of the forms:

(47) $$\begin{array}{c} \left\{\begin{array}{c} h_{1}\left(\omega \right)=Q_{+,:N}\left(\omega \right){\bar{h}}_{:N}\left(\omega \right)\\ h_{2}\left(\omega \right)=Q_{-,:N}\left(\omega \right){\bar{h}}_{:N}\left(\omega \right) \end{array}\right., \end{array}$$

where

(48) $$\begin{array}{c} {\bar{h}}_{:N}\left(\omega \right)=\left[\begin{array}{cccc} {\bar{H}}_{1}\left(\omega \right) & {\bar{H}}_{2}\left(\omega \right) & \cdots & {\bar{H}}_{N}\left(\omega \right) \end{array}\right]\neq 0 \end{array}$$

is a complex-valued filter of length *N*. For this class of orthogonal filters, the output IC for diffuse noise is

(49) $$\begin{array}{ccc} \gamma_{d}\left[h_{1}\left(\omega \right),h_{2}\left(\omega \right)\right] & = & \frac{{\bar{h}}_{:N}^{H}\left(\omega \right)\Lambda_{-,N}\left(\omega \right){\bar{h}}_{:N}\left(\omega \right)}{{\bar{h}}_{:N}^{H}\left(\omega \right)\Lambda_{+,N}\left(\omega \right){\bar{h}}_{:N}\left(\omega \right)}\\ & = & \gamma_{d}\left[{\bar{h}}_{:N}\left(\omega \right)\right], \end{array}$$

where

(50) $$\begin{array}{ccc} \Lambda_{-,N}\left(\omega \right) & = & \frac{1}{2}\mathrm{diag}\left[\lambda_{-,1}\left(\omega \right),\lambda_{-,2}\left(\omega \right),\ldots ,\lambda_{-,N}\left(\omega \right)\right], \end{array}$$

(51) $$\begin{array}{ccc} \Lambda_{+,N}\left(\omega \right) & = & \frac{1}{2}\mathrm{diag}\left[\lambda_{+,1}\left(\omega \right),\lambda_{+,2}\left(\omega \right),\ldots ,\lambda_{+,N}\left(\omega \right)\right]. \end{array}$$

It can be shown that

(52) $$\begin{array}{c} 1\geq \gamma \left[{\bar{h}}_{:1}\left(\omega \right)\right]\geq \gamma \left[{\bar{h}}_{:2}\left(\omega \right)\right]\geq \cdots \geq \gamma \left[{\bar{h}}_{:M}\left(\omega \right)\right]\geq 0. \end{array}$$

With (47), the binaural WNG, DF, and power beampattern can be expressed as, respectively,

(53) $$\begin{array}{c} W\left[{\bar{h}}_{:N}\left(\omega \right)\right]=\frac{{\bar{h}}_{:N}^{H}\left(\omega \right)C\left(\omega ,0\right)C^{H}\left(\omega ,0\right){\bar{h}}_{:N}\left(\omega \right)}{2{\bar{h}}_{:N}^{H}\left(\omega \right){\bar{h}}_{:N}\left(\omega \right)}, \end{array}$$

(54) $$\begin{array}{c} D\left[{\bar{h}}_{:N}\left(\omega \right)\right]=\frac{{\bar{h}}_{:N}^{H}\left(\omega \right)C\left(\omega ,0\right)C^{H}\left(\omega ,0\right){\bar{h}}_{:N}\left(\omega \right)}{2{\bar{h}}_{:N}^{H}\left(\omega \right)\Lambda_{+,N}\left(\omega \right){\bar{h}}_{:N}\left(\omega \right)}, \end{array}$$

and

(55) $$\begin{array}{c} {\left|B\left[{\bar{h}}_{:N}\left(\omega \right),\theta \right]\right|}^{2}=\frac{{\bar{h}}_{:N}^{H}\left(\omega \right)C\left(\omega ,\theta \right)C^{H}\left(\omega ,\theta \right){\bar{h}}_{:N}\left(\omega \right)}{2}, \end{array}$$

where

(56) $$\begin{array}{c} C\left(\omega ,\theta \right)=\left[\begin{array}{cc} Q_{+,:N}^{T}\left(\omega \right)d\left(\omega ,\theta \right) & Q_{-,:N}^{T}\left(\omega \right)d\left(\omega ,\theta \right) \end{array}\right] \end{array}$$

is a matrix of size N×2 with the distortionless constraint being

(57) $$\begin{array}{c} C^{H}\left(\omega ,0\right){\bar{h}}_{:N}\left(\omega \right)=1=\left[\begin{array}{c} 1\\ 1 \end{array}\right]. \end{array}$$

To fulfill this constraint, we must take N≥2.

Substituting (47) into (12) and using the distortionless constraint, the variance of $Z_{i}\left(\omega \right)$ becomes

(58) $$\begin{array}{c} \phi_{Z_{i}}\left(\omega \right)=\phi_{X}\left(\omega \right)+\phi_{V_{1}}\left(\omega \right)\times {\bar{h}}_{:N}^{H}\left(\omega \right)Q_{\pm ,:N}^{T}\left(\omega \right)\Gamma_{v}\left(\omega \right)Q_{\pm ,:N}\left(\omega \right){\bar{h}}_{:N}\left(\omega \right), \end{array}$$

where $Q_{\pm ,:N}\left(\omega \right)=Q_{+,:N}\left(\omega \right)$ for $\phi_{Z_{1}}\left(\omega \right)$ and $Q_{\pm ,:N}\left(\omega \right)=Q_{-,:N}\left(\omega \right)$ for $\phi_{Z_{2}}\left(\omega \right)$. In the case of diffuse-plus-white noise, i.e., $\Gamma_{v}\left(\omega \right)=\Gamma_{d}\left(\omega \right)+\alpha I_{2M}$, where α is a parameter that determines the relative level between the diffuse and white noises, (58) simplifies to

(59) $$\begin{array}{c} \phi_{Z_{i}}\left(\omega \right)=\phi_{X}\left(\omega \right)+\phi_{V_{1}}\left(\omega \right)\times \left[{\bar{h}}_{:N}^{H}\left(\omega \right)\Lambda_{+,N}\left(\omega \right){\bar{h}}_{:N}\left(\omega \right)+\alpha {\bar{h}}_{:N}^{H}\left(\omega \right){\bar{h}}_{:N}\left(\omega \right)\right], \end{array}$$

showing that $\phi_{Z_{1}}\left(\omega \right)=\phi_{Z_{2}}\left(\omega \right)$. Again, using (57), we find that the cross-correlation between $Z_{1}\left(\omega \right)$ and $Z_{2}\left(\omega \right)$ is

(60) $$\begin{array}{ccc} \phi_{Z_{1}Z_{2}}\left(\omega \right) & = & E\left[Z_{1}\left(\omega \right)Z_{2}^{*}\left(\omega \right)\right]\\ & = & \phi_{X}\left(\omega \right)+\phi_{V_{1}}\left(\omega \right){\bar{h}}_{:N}^{H}\left(\omega \right)Q_{+,:N}^{T}\left(\omega \right)\Gamma_{v}\left(\omega \right)Q_{-,:N}\left(\omega \right){\bar{h}}_{:N}\left(\omega \right), \end{array}$$

whose form for diffuse-plus-white noise is

(61) $$\begin{array}{c} \phi_{Z_{1}Z_{2}}\left(\omega \right)=\phi_{X}\left(\omega \right)+\phi_{V_{1}}\left(\omega \right){\bar{h}}_{:N}^{H}\left(\omega \right)\Lambda_{-,N}\left(\omega \right){\bar{h}}_{:N}\left(\omega \right), \end{array}$$

which, as expected, does not depend on the white noise. For $\Gamma_{v}\left(\omega \right)=\Gamma_{d}\left(\omega \right)+\alpha I_{2M}$, the output IC of the estimated signals is

(62) $$\begin{array}{cc} \gamma_{Z_{1}Z_{2}}\left(\omega \right)\; & =\frac{\phi_{Z_{1}Z_{2}}\left(\omega \right)}{\sqrt{\phi_{Z_{1}}\left(\omega \right)\phi_{Z_{2}}\left(\omega \right)}}\\ \; & =\frac{\mathrm{iSNR}\left(\omega \right)+{\bar{h}}_{:N}^{H}\left(\omega \right)\Lambda_{-,N}\left(\omega \right){\bar{h}}_{:N}\left(\omega \right)}{\mathrm{iSNR}\left(\omega \right)+{\bar{h}}_{:N}^{H}\left(\omega \right)\Lambda_{+,N}\left(\omega \right){\bar{h}}_{:N}\left(\omega \right)+\alpha {\bar{h}}_{:N}^{H}\left(\omega \right){\bar{h}}_{:N}\left(\omega \right)}. \end{array}$$

We deduce that for large input SNRs, the localization cues of the estimated signals depend mostly on the ones of the desired signal, while for low SNRs, they depend mostly on the ones of the diffuse-plus-white noise.

One possible binaural superdirective beamformer can be obtained by minimizing the sum of the filtered diffuse noise signals subject to the distortionless constraint in (57), i.e.,

(63) $$\begin{array}{ccc} & & \min_{{\bar{h}}_{:N}\left(\omega \right)}2{\bar{h}}_{:N}^{H}\left(\omega \right)\Lambda_{+,N}\left(\omega \right){\bar{h}}_{:N}\left(\omega \right)\\ & & \;\;\;\;\;\;\;\;\;\;\;s.\;t.\;\;\;{\bar{h}}_{:N}^{H}\left(\omega \right)C\left(\omega ,0\right)=1^{T}. \end{array}$$

We easily get

(64) $$\begin{array}{ccc} & & {\bar{h}}_{:N,\mathrm{BSD}}\left(\omega \right)=\Lambda_{+,N}^{-1}\left(\omega \right)C\left(\omega ,0\right){\left[C^{H}\left(\omega ,0\right)\Lambda_{+,N}^{-1}\left(\omega \right)C\left(\omega ,0\right)\right]}^{-1}1 \end{array}$$

and the corresponding binaural DF is

(65) $$\begin{array}{c} D\left[{\bar{h}}_{:N,\mathrm{BSD}}\left(\omega \right)\right]=\frac{1}{1^{T}{\left[C^{H}\left(\omega ,0\right)\Lambda_{+,N}^{-1}\left(\omega \right)C\left(\omega ,0\right)\right]}^{-1}1}. \end{array}$$

Therefore, the proposed binaural superdirective beamformer is

(66) $$\begin{array}{c} \left\{\begin{array}{c} h_{1,\mathrm{BSD}}\left(\omega \right)=Q_{+,:N}\left(\omega \right){\bar{h}}_{:N,\mathrm{BSD}}\left(\omega \right)\\ h_{2,\mathrm{BSD}}\left(\omega \right)=Q_{-,:N}\left(\omega \right){\bar{h}}_{:N,\mathrm{BSD}}\left(\omega \right) \end{array}\right.. \end{array}$$

Note that another form of the binaural superdirective beamformer can be derived by maximizing the binaural DF in (18) subject to the distortionless constraints, which will be left to the reader’s investigation to make the paper concise.

## 6. Experiments and Analysis

In this section, we study the performance of the developed binaural heterophasic superdirective beamforming method and compare it to the monaural superdirective beamformer through experiments. For fair comparison, the orders of the binaural heterophasic and the monaural superdirective beamformers are set to the same number so the DF of the two beamformers would be similar.

### 6.1. Performance Analysis

**Figure 3:**
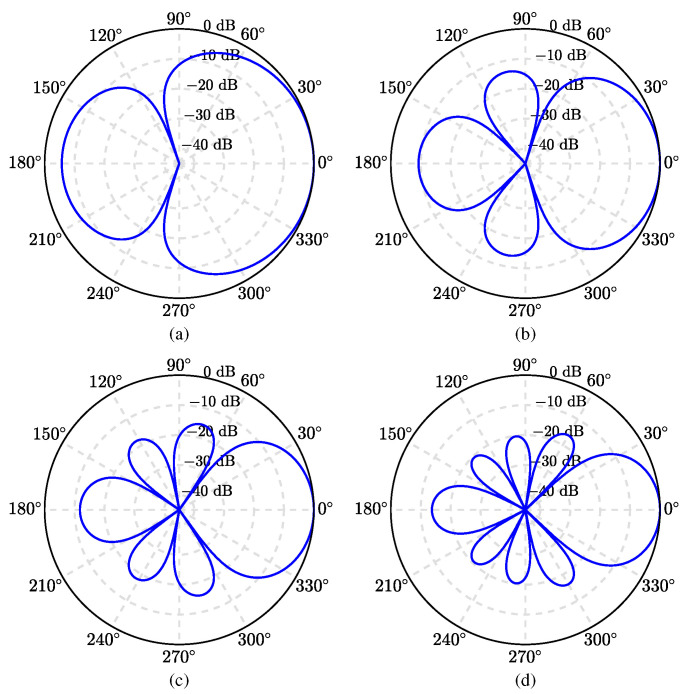
Beampatterns of the binaural heterophasic superdirective beamformer with various numbers of microphones: (**a**) M=6, (**b**) M=8, (**c**) M=10, and (**d**) M=12. Conditions of simulation: f=1 kHz, δ=1 cm, and N=M/2.

**Figure 4:**
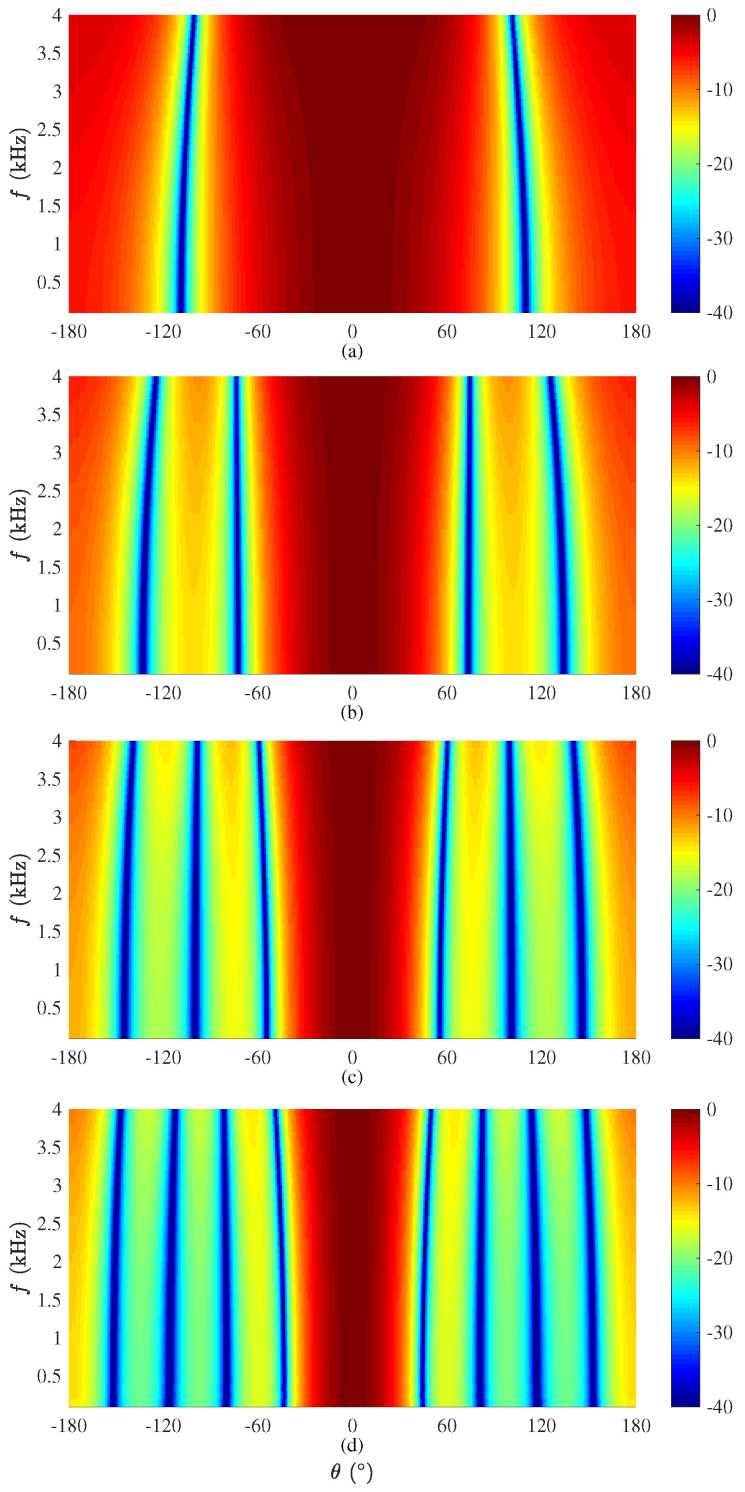
Beampatterns of the binaural heterophasic superdirective beamformer as a function of the frequency with different numbers of microphones: (**a**) M=6, (**b**) M=8, (**c**) M=10, and (**d**) M=12. Conditions of simulation: δ=1.0 cm and N=M/2.

We first evaluate the beampattern (given in (55)) of the binaural superdirective beamformer. A ULA is used with an interelement spacing equal to 1 cm. The beampatterns of the derived beamformer are plotted in Figure 3, where M=6,8,10,12, and N=M/2, at f=1 kHz. Note that, given a ULA with *M* microphone sensors, one can design binaural superdirective beamformers of order from 1 to M/2. When the order increases, the DF becomes larger but the WNG becomes smaller. In this work, we only show the case with N=M/2 in the simulation for maximum DF. Two-dimensional (2D) plots of the corresponding beampatterns are shown in Figure 4; one can observe that, in all cases, they are almost frequency invariant.

**Figure 5:**
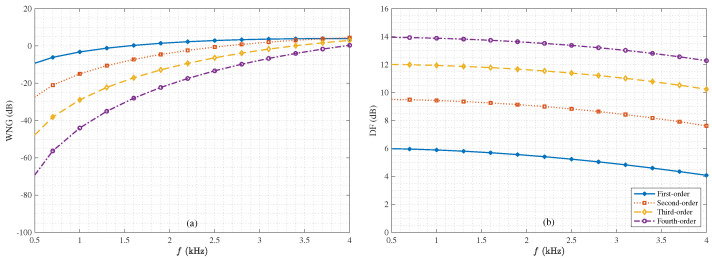
WNGs and DFs of the 1st-, 2nd-, 3rd-, and 4th-order binaural heterophasic superdirective beamformers versus frequency: (**a**) WNGs and (**b**) DFs. The sensor spacing: δ=1 cm.

**Figure 6:**
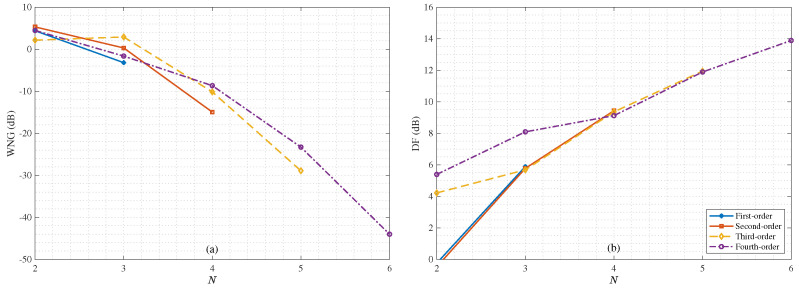
WNGs and DFs of the 1st-, 2nd-, 3rd-, and 4th-order binaural heterophasic superdirective beamformers versus parameter *N*: (**a**) WNGs and (**b**) DFs. Conditions of simulation: f=1 kHz and δ=1 cm.

Next, We study the performance of the binaural heterophasic superdirective beamformer in terms of WNG and DF, according to (53) and (54), respectively. The results for the WNG and DF are plotted in Figure 5, where the first-, second-, third-, and fourth-order binaural superdirective beamformers are designed with M=6,8,10,12, respectively (this is the basic requirement for the design of the binaural heterophasic superdirective beamformer as shown in Section 5). One can see that the WNGs of binaural superdirective beamformer decrease with the order while the DFs of beamformers increase with the order. Besides, for each order, the DF does not change much with frequency, which is an important property for processing broadband signals like speech. Figure 6 plots the WNGs and DFs of the binaural superdirective beamformers versus parameter *N*, where 2≤N≤M/2. This parameter is introduced to gain flexibility for achieving compromise between WNG and DF. As seen, the DF increases while the WNG decreases with the increase of the value of *N*. In practice, one can tune the parameter *N* according to the application requirement.

**Figure 7:**
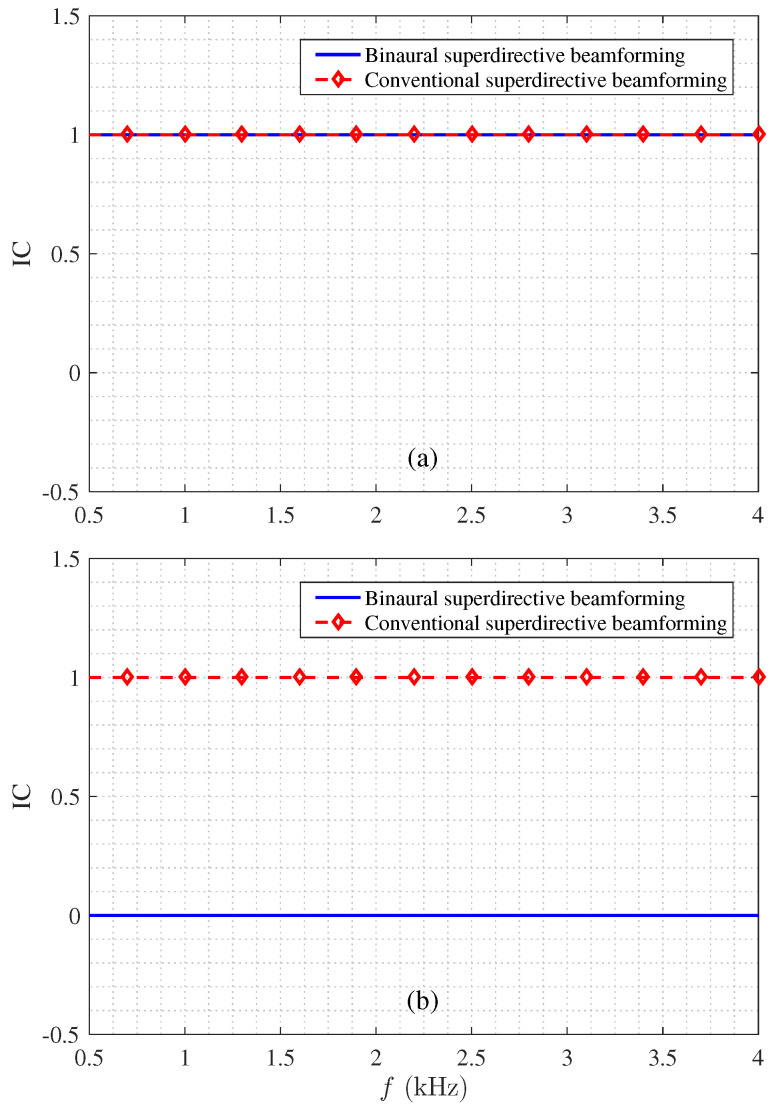
Magnitude of the output ICs: (**a**) diffuse noise and (**b**) white noise. Conditions of simulation: M=8 for the binaural superdirective beamformer, M=3 for the conventional superdirective beamformer, and δ=1 cm. Note that the output IC of the binaural superdirective beamformer in diffuse noise is frequency-dependent; it approaches 1 under 4 kHz but decreases as the frequency increases.

Next, we study the ICs of the binaural heterophasic and conventional superdirective beamformers under the same conditions according to (23) and (24). Figure 7 plots the output ICs of both beamformers as a function of frequency in white and diffuse noises, respectively. As seen, in the diffuse noise case, the ICs of both beamformers are equal to one within the studied frequency range; in the white noise case, the IC of the binaural superdirective beamformer is equal to zero while for the conventional superdirective beamformer it is equal to one. This means that in the two output signals of the binaural superdirective beamformer, the speech signal is completely coherent, while the white noise is completely incoherent; so, the output signals correspond to the heterophasic case as discussed in Section 1, in which the speech and white noise can be regarded as two separate direction sources in space.

**Figure 8:**
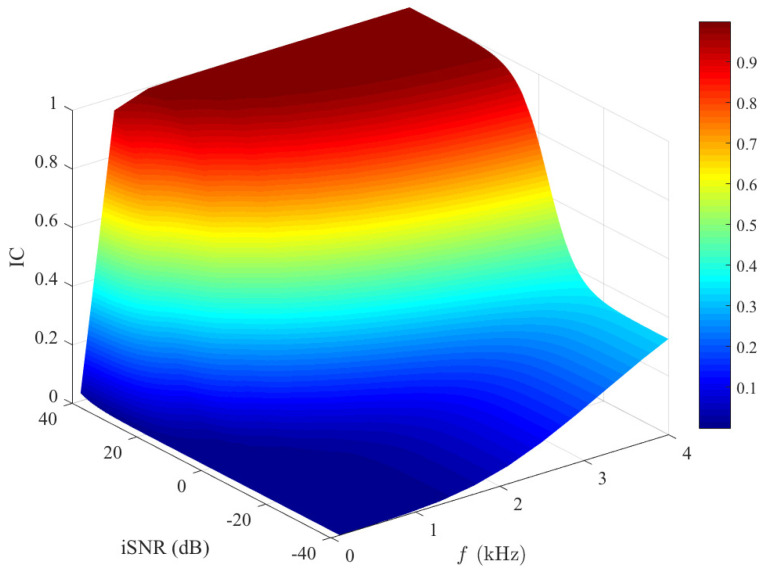
Output IC of the estimated signals (see (62)) as a function of frequency and input SNR. Conditions of simulation: M=8, N=4, α=1, and δ=1 cm.

Figure 8 plots the IC magnitude of the outputs of the binaural heterophasic superdirective beamformer, which is given in (62), versus frequency in different input SNR conditions. One can see that this IC increases with frequency. This is due to the fact that white noise amplification mainly happens at low frequencies. The output IC in white noise is zero, causing the low-frequency output IC of the entire signal to approach zero. This shows the impact of white noise amplification from the perspective of the output IC. For a fixed frequency, it is seen that the output IC increases with the input SNR, and it approaches one at a high input SNR. This can be easily explained: as the input SNR increases, the desired signal component dominates the beamforming output. The output IC of the desired signal is one, so the output IC of the two output signals in this condition also approaches one. Consequently, one can conclude that, for high input SNRs, the localization cues of the estimated signals depend mostly on the ones of the desired signal, while at low SNRs, they depend mostly on the ones of the noise.

### 6.2. Experiments in Real Environments

**Figure 9:**
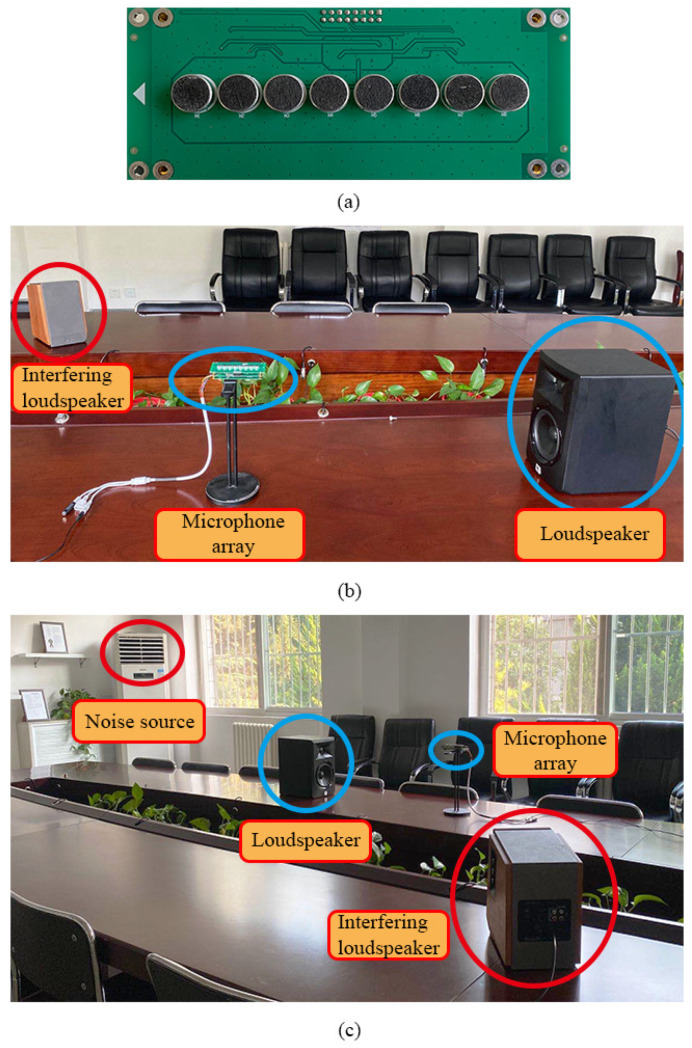
A photo of the designed array and the experimental setup for evaluating the binaural and conventional superdirective beamformers: (**a**) photo of the designed eight-microphone array, (**b**) a close view photo of the experimental setup, and (**c**) a wide angle photo of the experimental setup.

In this subsection, we evaluate the performance of the proposed binaural heterophasic superdirective beamformer in real acoustic environments. The experiments were conducted in a 10.5×6×2.8 m conference room. A ULA is used, which consists of 8 microphones, where the elements spacing is δ=1.1 cm. The SNR of the microphones is 60 dB(A). A photo of the designed array and the experimental setup are shown in Figure 9. To make the experiments repeatable, we first used the microphone array to record sound signals from a loudspeaker located in the ULA’s endfire direction. Then, both the conventional and binaural superdirective beamformers were implemented to get the outputs.

**Figure 10:**
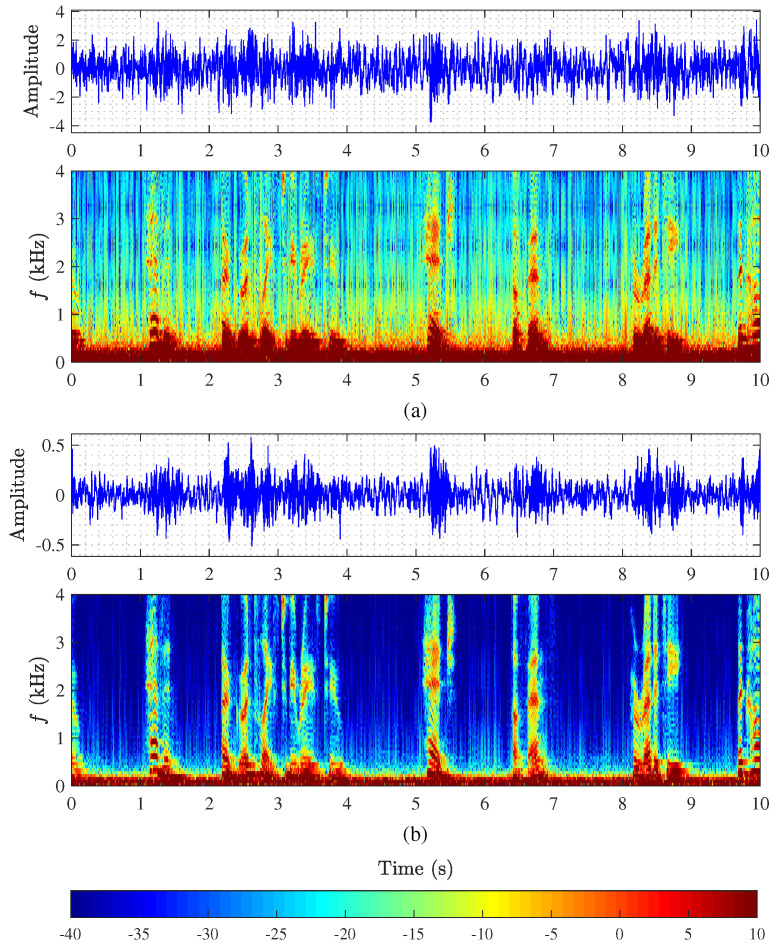
Monaural and binaural superdirective beamformers in a conference room: (**a**) output of the 2nd-order monaural superdirective beamformer and its spectrogram with M=3 and δ=1.1 cm, and (**b**) output of the 2nd-order binaural superdirective beamformer and its spectrogram with M=8 and δ=1.1 cm.

Figure 10 plots the time-domain signals and their spectrograms of the output signals of the conventional and binaural superdirective beamformers. It is clearly seen that the outputs of both beamformers suffer from serious white noise amplification, where the desired signal is almost covered by the white noise. It is also seen from spectrograms that white noise amplification mainly occurs at low frequencies.

**Figure 11:**
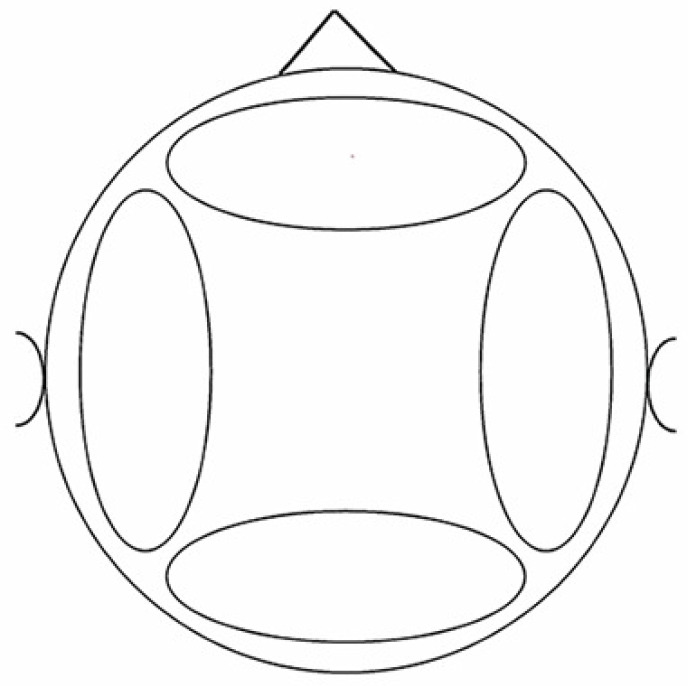
An illustration of the auditory map for subjects (horizontal-plane). During the test, the subjects were asked to mark the areas according to the sound source location they heard through headphones.

As emphasized previously, the main advantage of the proposed binaural superdirective beamforming method is to have the human binaural auditory system to better separate the signal of interest from white noise after beamforming. To confirm this, we performed some subjective listening experiments. Firstly, we obtained a series of output signals of the implemented conventional and binaural superdirective beamformers. Specifically, we extracted seven audio clips from the “Voice of America” with each of length of 20 s. After playing and recording through the loudspeaker and the microphone array shown in Figure 9, we use the two kind of beamformers to perform superdirective beamforming to obtain the output signals. Then, five subjects were asked to listen to the output signals and draw up the zones of the sound source and white noise on the horizontal plane within a predesigned circle. Finally, we extracted the images sketched by each subject and averaged them to get the experimental results (note that in these experiments, we selected five subjects from the CIAIC–Center of Intelligent Acoustics and Immersive Communications, who are experienced in acoustic analysis and can clearly distinguish auditory events). Here we only provide four different zones, i.e., front, back, left, and right sides, to ask the subjects to choose, where an illustration of the test is shown in Figure 11.

**Figure 12:**
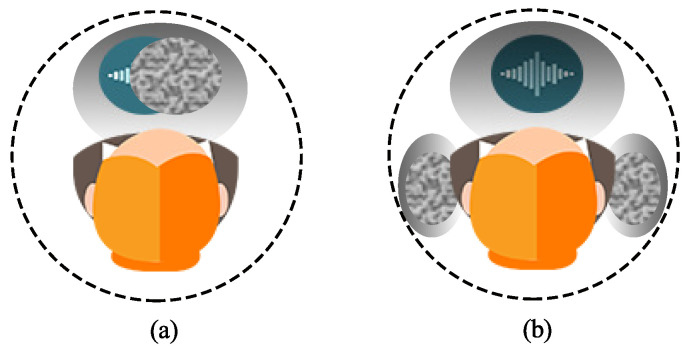
The average auditory map marked by the listening subjects: (**a**) monaural superdirective beamformer and (**b**) binaural superdirective beamformer. The blue waves refer to the region in which the desired speech is heard, and the region filled with disorderly dots refer to the region in which the white noise is heard. Conditions of experiment: M=8, N=4, and δ=1.1 cm.

Figure 12 presents the auditory maps averaged from the five subjects from the horizontal plane. As can be seen, for the conventional superdirective beamformer, all signals (desired signal plus white noise) are perceived to be in the middle of the head, which corresponds to the homophasic case. Oppositely, for the binaural superdirective beamforming, the signal of interest is perceived to be in the median plane of the head while white noise is located in each side of the ear, which corresponds to the heterophasic case. As discussed previously and summarized in Table 1, the speech intelligibility in heterophasic case is higher than the homophasic case (approximately 4 dB higher [49]). Consequently, the proposed binaural superdirective beamformer has better intelligibility than the conventional one.

## 7. Conclusions

In this paper, we addressed the problem of superdirective beamforming with small-spacing microphone arrays. While it can achieve the maximum spatial gain to suppress acoustic noise, the traditional superdirective beamformer suffers from white noise amplification, which is particularly serious at low frequencies. Many methods were developed in the literature to deal with this problem, but they all pay a price of sacrificing the DF and the resulting beamformers may no longer be superdirective. Motivated by studies and facts in psychoacoustics, we developed in this paper a binaural heterophasic superdirective beamformer, which consists of two sub-beamforming filters, each for one of the binaural channels. These two sub-beamformers are constrained to be orthogonal to each other to minimize the IC of the white noise components in the binaural outputs while maximize the IC of the diffuse noise components. As a result, the signal of interest in the binaural superdirective beamformer’s outputs is in phase while the white noise is random in phase, so that the human auditory system is able to more easily separate the acoustic signal of interest from white noise by listening to the outputs of the proposed beamformer. Simulations and experiments were carried out to evaluate the performance of the proposed binaural superdirective beamformer. The results corroborate with the theoretical analysis and confirm that the binaural superdirective beamforming corresponds to the heterophasic case studied in psychoacoustics. Based on the listening tests shown in the psychoacoustic study, one can conclude that the improvement in intelligibility is expected to be 4 dB at low SNR conditions in accordance with the psychoacoustic experiments verified in the literature.

## Figures and Tables

**Table 1:** Different scenarios for intelligibility study based on phase relationship between speech and noise [49].

Scenario	Speech	Noise	Class
1	Out of phase	In phase	Antiphasic
2	In phase	Out of phase	Antiphasic
3	In phase	Random phase	Heterophasic
4	Out of phase	Random phase	Heterophasic
5	In phase	In phase	Homophasic
6	Out of phase	Out of phase	Homophasic

## Data Availability

Data sharing not applicable.
